# Navigating Diagnostic Ambiguity in Palliative Pediatric Neuro-Oncology: A Conceptual Framework for Suspected Shunt Malfunction and Refractory Hyponatremia

**DOI:** 10.7759/cureus.106956

**Published:** 2026-04-13

**Authors:** Olivia R Kaufman, Samira Haberman, Christopher M Ahmad, Miriana Youkhana

**Affiliations:** 1 Medicine, Kansas City University, Joplin, USA; 2 Family Medicine, Rosalind Franklin University of Medical Sciences, Chicago, USA

**Keywords:** medulloblastoma, obstructive hydrocephalus, pediatric neuro-oncology, pediatric palliative care, shunt malfunction

## Abstract

Management of suspected shunt malfunction in pediatric patients with terminal brain tumors presents a complex clinical and ethical dilemma. Differentiating mechanical shunt failure from disease progression or metabolic derangement is often radiographically indeterminate, while invasive diagnostics may conflict with palliative goals of care. We present the case of a five-year-old male with relapsed medulloblastoma and cerebrospinal fluid (CSF) dissemination who developed progressive ventriculomegaly and refractory hyponatremia (serum sodium nadir: 120 mEq/L). The clinical course was complicated by the overlap of potential etiologies: shunt failure, syndrome of inappropriate antidiuretic hormone secretion (SIADH) versus cerebral salt wasting (CSW), and terminal disease progression. This report details the diagnostic challenges encountered and proposes a conceptual framework for structuring clinical decision-making in the setting of diagnostic uncertainty. This framework prioritizes three parallel workstreams: (1) imaging trend analysis (emphasizing delta over static findings), (2) metabolic profiling (using osmolality to guide risk stratification), and (3) continuous goals-of-care realignment. In the palliative setting, definitive diagnosis of shunt failure is often unattainable. We suggest that, in selected palliative contexts, management may benefit from shifting away from the pursuit of diagnostic certainty toward a risk-adapted, goal-concordant approach.

## Introduction

Medulloblastoma is the most common malignant brain tumor in children, typically located in the posterior fossa [1]. Standard treatment for children over age three involves maximal surgical resection, craniospinal irradiation, and chemotherapy [2,3]. While initial survival rates have improved, relapse, especially with CSF dissemination, remains fatal in most cases [2].

In neuro-oncology patients, hyponatremia is often attributed to syndrome of inappropriate antidiuretic hormone secretion (SIADH) or cerebral salt wasting (CSW). Although both may present with low serum sodium, low plasma osmolality, and inappropriately concentrated urine, SIADH is typically associated with euvolemia and is commonly managed with fluid restriction, whereas CSW is associated with renal sodium loss and relative hypovolemia, often requiring salt and volume repletion. Distinguishing the two at the bedside can be difficult, particularly in critically ill children with complex neurologic disease.

Similarly, progressive ventricular enlargement does not always indicate shunt obstruction. In heavily treated pediatric brain tumor patients, ventricular enlargement may also reflect ex vacuo change related to parenchymal loss after surgery, radiation, or diffuse disease progression. As a result, serial neuroimaging may raise concern for shunt malfunction without definitively establishing that mechanical failure is the primary driver of neurologic decline.

In the palliative pediatric neuro-oncology setting, this challenge is further complicated by hydrocephalus, which is common in posterior fossa tumors and often necessitates ventriculoperitoneal (VP) shunting [4-6]. However, in heavily treated children, persistent ventriculomegaly may reflect either high-pressure obstructive hydrocephalus or ex vacuo enlargement related to treatment-associated parenchymal loss. Extensive postsurgical and post-radiation changes can therefore make it difficult to determine whether ventricular enlargement reflects true mechanical shunt failure or disease-related ventricular remodeling.

This uncertainty is often compounded by refractory hyponatremia, particularly when the differential includes SIADH and CSW, which carry opposing management implications [7].

We present a case of relapsed medulloblastoma complicated by both suspected shunt failure and severe hyponatremia. This report presents a conceptual framework intended to help structure management when definitive diagnosis is not possible, and invasive interventions must be weighed against palliative goals of care [8].

## Case presentation

History and initial diagnosis

**Figure 1:**
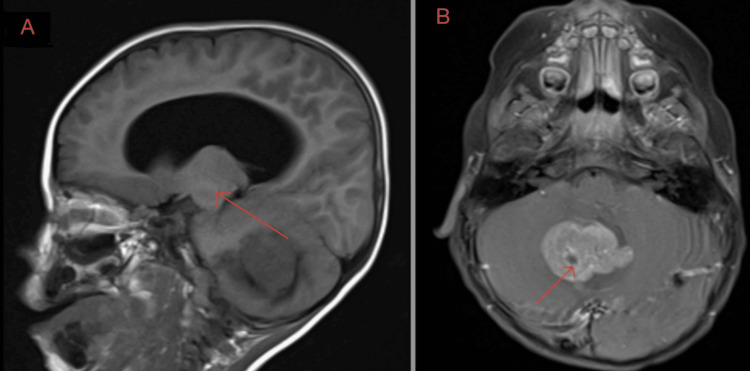
MRI demonstrating posterior fossa mass with obstructive hydrocephalus (A) Sagittal MRI showing a posterior fossa mass arising from the cerebellar vermis (red arrows) with compression of the fourth ventricle. (B) Axial MRI demonstrating the same lesion (red arrows) with associated ventricular enlargement consistent with obstructive hydrocephalus. These findings were identified during the patient’s initial diagnostic workup. MRI: Magnetic resonance imaging.

A 27-month-old male with no significant prior medical history presented in August 2017 following two weeks of progressive gait instability, truncal ataxia, and forceful vomiting. Initial physical examination revealed papilledema and significant lethargy, raising immediate concern for increased intracranial pressure. Neuroimaging via MRI confirmed a 4.3-cm midline posterior fossa mass arising from the cerebellar vermis, which was severely compressing the fourth ventricle and causing significant obstructive hydrocephalus (Figure 1). These initial findings established the structural basis for obstructive hydrocephalus early in the disease course and provided an important baseline against which later ventricular changes were interpreted.

The patient underwent emergent right-sided external ventricular drain (EVD) placement, followed by subtotal craniotomy and tumor resection on August 28, 2017. Histopathologic evaluation and molecular profiling confirmed a WHO Grade IV classic medulloblastoma, consistent with integrated diagnostic criteria that combine histologic subtype with molecular classification in contemporary medulloblastoma diagnosis [9]. His early postoperative course was notably complicated by posterior fossa syndrome, characterized by speech apraxia, global hypotonia, and emotional lability.

Therapeutic evolution and relapse

**Figure 2:**
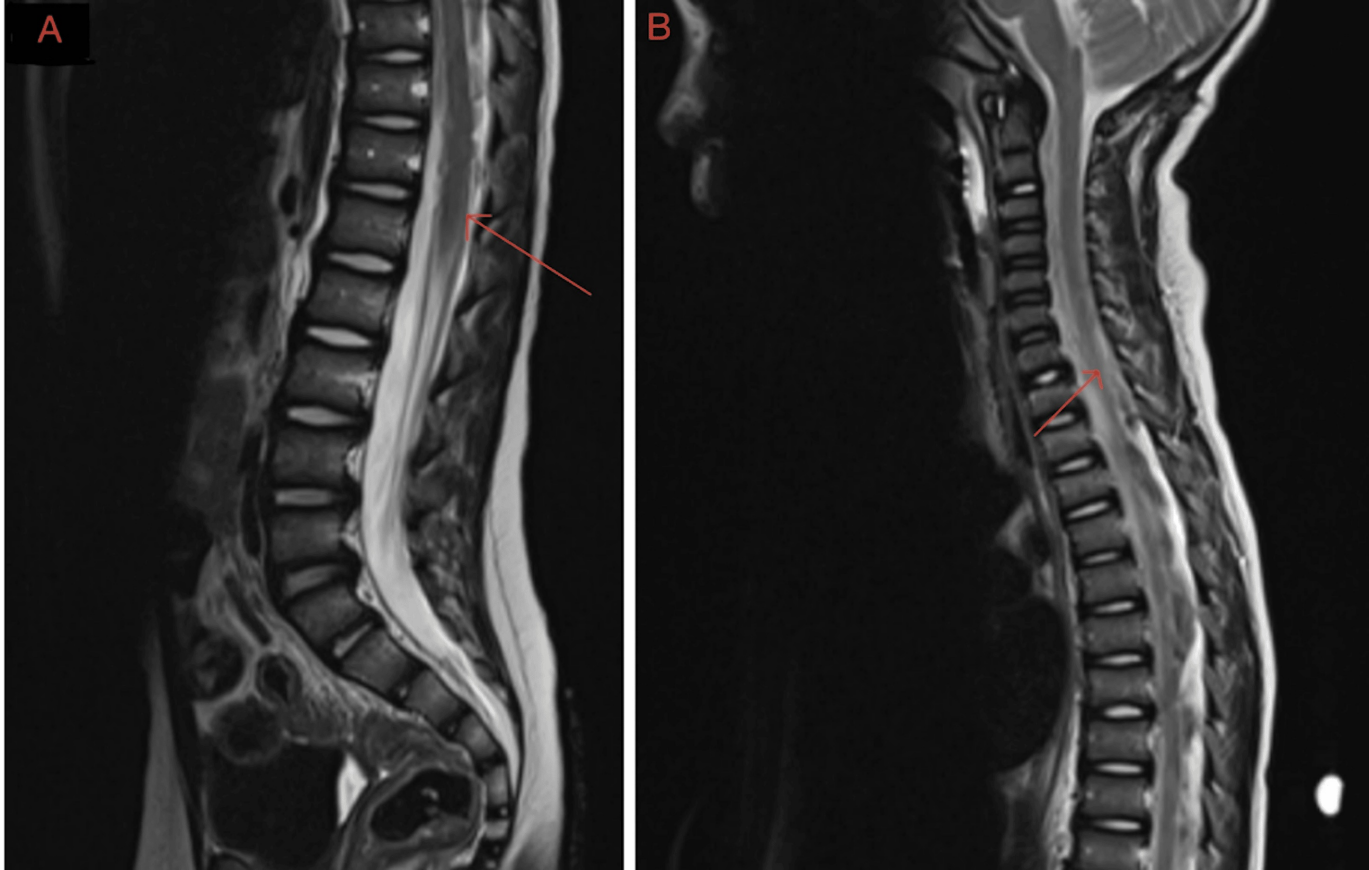
Sagittal MRI demonstrating leptomeningeal dissemination (red arrows) The image shows recurrence via leptomeningeal dissemination throughout the spinal column (red arrows). Imaging was warranted due to a new onset of vomiting and ataxia. MRI: Magnetic resonance imaging.

Following surgical stabilization, the patient began an intensive adjuvant chemotherapy regimen (ACNS0334, Regimen B). He completed this initial protocol in March 2018; however, approximately 10 months later, he experienced a clinical recurrence marked by a return of ataxia and vomiting. Surveillance MRI confirmed disease progression with leptomeningeal dissemination and multiple nodular drop metastases along the neuraxis (Figure 2). These findings reframed the patient’s prognosis and increased concern that subsequent neurologic decline would reflect both progressive disease burden and secondary CSF flow disruption rather than a purely focal recurrence.

The patient was subsequently referred for craniospinal irradiation (CSI) with proton therapy at an external facility. Shortly after completion of radiation, imaging revealed a new area of recurrence along the left optic nerve. In response to this refractory progression, the patient was transitioned to a MEMMAT (Medulloblastoma European Multitarget Metronomic Anti-angiogenic Trial)-based regimen, a metronomic anti-angiogenic salvage strategy used in recurrent medulloblastoma [10].

Clinical deterioration and salvage attempts

Two months after the diagnosis of relapse, the patient was admitted to the pediatric intensive care unit (PICU) following acute clinical deterioration. His presentation was characterized by profound lethargy, sialorrhea (drooling), and an altered mental status. Due to the failure of the MEMMAT protocol, he was transitioned to the ACNS0821 regimen. This treatment continued until early 2020, when he presented to the emergency department with high fevers, worsening somnolence, and new-onset seizure activity. Upon this final admission, his Glasgow Coma Scale (GCS) was 10, and he exhibited signs of significant neurological failure.

The diagnostic dilemma: hydrocephalus vs. atrophy

**Figure 3:**
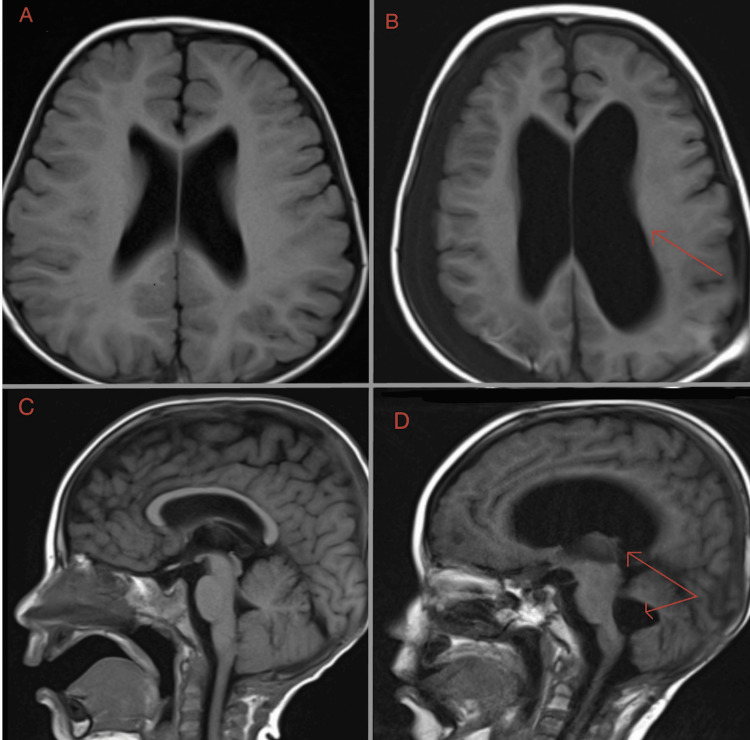
Serial neuroimaging demonstrating progressive ventriculomegaly in a child with suspected shunt malfunction (A) Baseline axial MRI following tumor resection demonstrating normal ventricular caliber. (B) Axial MRI during admission demonstrating marked dilation of the lateral and third ventricles consistent with acute ventriculomegaly (red arrow). (C) Baseline sagittal MRI demonstrating normal ventricular anatomy and third ventricular size. (D) Sagittal MRI obtained during clinical deterioration demonstrating enlargement of the ventricular system, particularly the third ventricle (red arrow). MRI: Magnetic resonance imaging.

During his final weeks, serial neuroimaging demonstrated a progressive enlargement of the lateral and third ventricles (Figure 3). Serial comparison demonstrated interval ventricular enlargement; however, the imaging remained insufficient to distinguish true high-pressure shunt failure from ex vacuo enlargement in the setting of cumulative treatment-related brain injury. This created a significant diagnostic dilemma for the neurosurgical team. While the ventriculomegaly was progressive, the distinction between high-pressure obstructive hydrocephalus, which would require surgical shunt revision, and ventriculomegaly ex vacuo (secondary to brain tissue atrophy from intensive radiation and chemotherapy) was obscured. The extensive history of prior surgical resections and the presence of significant tumor burden at the base of the brain complicated the interpretation of whether a mechanical shunt failure was the primary driver of his decline.

Metabolic crisis and diagnostic overlap

Trends in sodium levels, plasma osmolality, urine osmolality, and CRP were followed throughout the course of admission (Table 1). Table 1 illustrates persistent hypotonic hyponatremia with inappropriately concentrated urine and incomplete correction over time, reinforcing that the metabolic disturbance remained clinically relevant even as the neurologic picture worsened. Concurrently, the patient developed persistent and clinically significant metabolic derangement. His admission serum sodium was 123 mEq/L and continued to trend downward. Initial management for suspected SIADH, including fluid restriction, failed to achieve correction. Further diagnostic workup revealed a plasma osmolality of 238 mOsm/kg and an inappropriately elevated urine osmolality of 850 mOsm/kg, with high urinary sodium excretion. The laboratory profile was compatible with hypotonic hyponatremia and inappropriately concentrated urine, but definitive phenotyping remained limited because the clinical picture did not cleanly fit a single syndrome. Persistent hyponatremia despite initial restriction-based management raised concern that isolated SIADH was insufficient to explain the course, while incomplete volume-status data limited confident diagnosis of CSW.

**Table 1:** Summary of electrolyte and osmolality findings during hospitalization SIADH: Syndrome of inappropriate antidiuretic hormone; CSW: Cerebral salt wasting; CRP: C-reactive protein.

Test	Reference Range	Day 1	Day 3	Day 5	Day 7	Day 10
Serum sodium (mEq/L)	135–145	123	121	120	122	123
Plasma osmolality (mOsm/kg)	275–295	241	-	238	-	240
Urine osmolality (mOsm/kg)	300–900	830	-	850	-	800
CRP (mg/dL)	<1.0	7.6	6.9	5.4	4.8	5.0
Notable findings	-	Initial hyponatremia; concern for SIADH vs CSW	Persistent hyponatremia despite supportive management	Repeat osmolality studies; refractory trend	Continued monitoring during clinical decline	Ongoing laboratory-guided electrolyte management

This biochemical profile raised a second dilemma: the differential between SIADH and CSW. These two conditions require diametrically opposed management strategies, such as fluid restriction for SIADH versus aggressive fluid and salt replacement for CSW. Attempts to correct the sodium via hypertonic saline and salt supplementation provided only transient increases, with sodium levels frequently plateauing between 120 and 122 mEq/L, suggesting a profound disruption of central salt-handling mechanisms.

Decision-making and outcome

The clinical team, comprising neurology, neurosurgery, oncology, and critical care, faced a critical management decision: the coexistence of progressive ventriculomegaly and metabolic instability meant that any neurosurgical intervention carried a prohibitive risk. There was no clear evidence that a shunt revision would lead to a restoration of meaningful neurologic interaction, given the advanced stage of his leptomeningeal disease.

In consensus with the family, the decision was made to prioritize comfort and dignity over non-beneficial surgical interventions. The patient was transitioned to a palliative care pathway, focusing on the minimization of suffering. He remained hospitalized under these comfort measures until his death, ending a nearly three-year clinical course defined by aggressive Grade IV disease and complex treatment-related sequelae.

## Discussion

**Figure 4:**
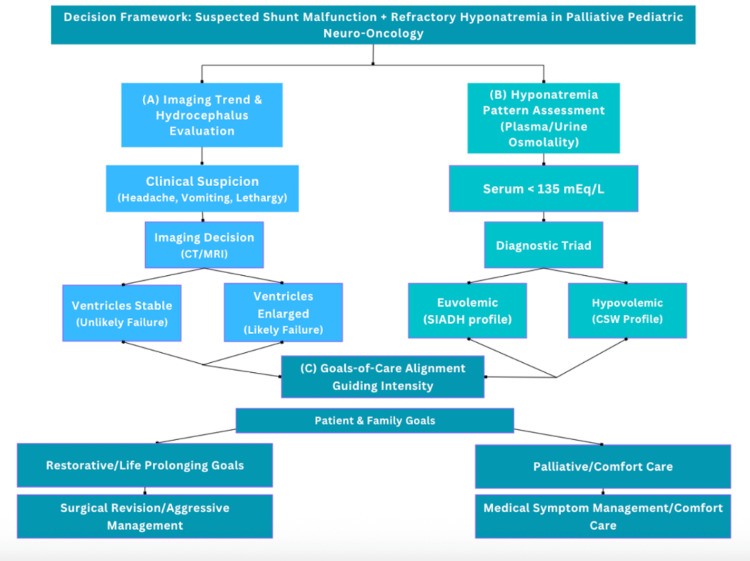
A proposed decision framework for clinical ambiguity in palliative neuro-oncology The schematic illustrates three parallel assessment streams guiding management decisions: (A) imaging trend analysis emphasizing the rate of ventricular change rather than static ventriculomegaly, (B) metabolic profiling to differentiate syndrome of inappropriate antidiuretic hormone secretion (SIADH) from cerebral salt wasting (CSW), and (C) goals-of-care alignment. The schematic illustrates how patient-centered goals may influence whether management leans toward surgical intervention (e.g., shunt revision) or palliative symptom-directed care in the setting of overlapping imaging and metabolic abnormalities.

The management of suspected shunt malfunction in pediatric neuro-oncology is frequently complicated by the "post-treatment effect," the permanent alteration of ventricular architecture by prior resection and radiation [1]. In the palliative setting, this diagnostic ambiguity is often compounded by metabolic derangements, creating a "neuro-metabolic crisis" where the optimal path between aggressive intervention and comfort care is unclear. To help structure this complexity, we present a three-stream conceptual framework (Figure 4).

The imaging stream: the "delta" vs. static findings

Differentiating mechanical shunt failure from ex vacuo ventriculomegaly is a well-documented challenge in patients with prior posterior fossa syndrome [4,11]. In patients with cerebral atrophy, ventricles may appear enlarged relative to healthy controls without indicating elevated intracranial pressure (ICP) [12]. In this context, reliance on a single static image may overestimate the likelihood of mechanical shunt failure [4,5]. Greater emphasis may therefore be placed on interval change across serial scans rather than on static ventricular size alone. However, even when interval enlargement is present, clinicians must still weigh whether shunt-directed intervention is likely to offer meaningful benefit. In disseminated disease, "trapped ventricle" phenomena or compartmentalization caused by leptomeningeal coating may render simple shunt revision ineffective, exposing the child to surgical pain with no functional benefit [5].

The metabolic stream: phenotyping the hyponatremia

Refractory hyponatremia is a common but perilous complication in CNS malignancies [7,13,14]. The differential diagnosis primarily lies between SIADH and CSW. This distinction is clinically important because SIADH is typically managed with fluid restriction, whereas CSW generally requires sodium and volume repletion [14]. Misclassification may worsen neurologic or hemodynamic instability [13,15]. In this case, the patient’s mixed picture (high urine osmolality but refractory to restriction) suggested a complex neuro-endocrine dysfunction common in terminal brainstem compression. In practice, this degree of metabolic instability may further increase the perceived risk of anesthesia and invasive intervention.

The decision gatekeeper: goals of care

Arguably, the most important component of this conceptual framework is goals-of-care alignment. In a restorative setting, the combination of progressive ventriculomegaly and neurologic decline would typically prompt consideration of EVD placement or shunt exploration. However, in the context of relapsed, disseminated medulloblastoma, the invasiveness of the procedure must be calibrated against the expected quality of life [15,16]. Importantly, neither the imaging findings nor the biochemical abnormalities were interpreted in isolation; rather, their combined uncertainty increased the perceived risk of invasive intervention while decreasing confidence that shunt revision would restore meaningful neurologic function. Literature suggests that while palliative shunting can improve symptom control (e.g., headache, vomiting), it rarely reverses deficits caused by tumor progression [15]. In this case, this conceptual framework helped organize the competing clinical considerations and supported a care plan that remained consistent with the family’s preference for comfort-focused, non-invasive end-of-life care [17]. Close collaboration among neurology, oncology, critical care, and palliative care was essential in determining the most patient-centered path forward. Because urine sodium values, complete volume-status characterization, and invasive shunt assessment were not available or not pursued, the competing diagnoses remained probabilistic rather than confirmatory.

Limitations

This report reflects a single pediatric case and is therefore limited in generalizability. The proposed three-stream framework was derived retrospectively from one clinical course and should be interpreted as conceptual and hypothesis-generating rather than validated or prescriptive. Definitive diagnostic confirmation was not pursued because management was appropriately shaped by established goals of care. Urine sodium measurements, formal hemodynamic volume-status assessments, detailed shunt valve parameters, and complete shunt revision history were not consistently available, limiting confident differentiation between SIADH, cerebral salt wasting, and mechanical shunt failure. No invasive intracranial pressure monitoring or shunt interrogation was performed, and sustained therapeutic trials to distinguish competing etiologies were not feasible. In addition, extensive postsurgical and post-radiation changes limited radiographic interpretability. The clinical trajectory itself is not biologically rare; the contribution of this report lies primarily in the structured framing of decision-making under diagnostic uncertainty in a palliative pediatric neuro-oncology setting.

## Conclusions

This report highlights the complexity of managing suspected shunt malfunction and refractory hyponatremia in children with advanced medulloblastoma, where diagnostic clarity is frequently limited by prior neurosurgical intervention and progressive disease. In such settings, ventriculomegaly and electrolyte disturbances often arise from overlapping etiologies, making definitive differentiation between mechanical shunt failure and terminal disease progression radiographically and clinically uncertain. Rather than serving as absolute diagnostic endpoints, imaging and biochemical findings should be interpreted as probabilistic signals within a broader clinical context. The proposed framework should therefore be interpreted as a conceptual and hypothesis-generating aid derived from this case rather than as a validated algorithm for universal application.

In palliative pediatric neuro-oncology, the continued pursuit of diagnostic certainty may introduce additional harm without proportional benefit. This case suggests the potential value of a risk-adapted approach that integrates imaging trends, metabolic profiling, and, most critically, goals-of-care alignment. Early involvement of palliative care is essential to guide high-stakes decision-making in these neuro-metabolic crises. Arguably, the most important component of this conceptual framework is goals-of-care alignment.
